# Effectiveness of Chinese Herbal Medicine in Postoperative Fatigue Syndrome Following Total Joint Arthroplasty or Hip Fracture Surgery: Evidence from Randomized Controlled Trials

**DOI:** 10.2174/0113862073258802231107060433

**Published:** 2023-11-29

**Authors:** Jinlong Zhao, Guanghui Zhou, Zhongsheng Wang, Guihong Liang, Xingde Wei, Bangxin Sha, Weiyi Yang, Jun Liu, Hongyun Chen

**Affiliations:** 1 The Second Clinical College of Guangzhou University of Chinese Medicine, Guangzhou, 510405, China;; 2 Medical College of Acupuncture-Moxibustion and Rehabilitation of Guangzhou University of Chinese Medicine, Guangzhou, China;; 3 The Second Affiliated Hospital of Guangzhou University of Chinese Medicine, Guangzhou, 510120, China;; 4 The Fifth Clinical College of Guangzhou University of Chinese Medicine, Guangzhou, 510405, China;; 5 The Research Team on Bone and Joint Degeneration and Injury of Guangdong Provincial Academy of Chinese Medical Sciences, Guangzhou 510120, China;; 6 Guangdong Second Traditional Chinese Medicine Hospital (Guangdong Province Engineering Technology Research Institute of Traditional Chinese Medicine), Guangzhou, 510095, China

**Keywords:** Chinese herbal medicine, postoperative fatigue syndrome, total joint arthroplasty, hip fracture surgery

## Abstract

**Background:**

There is no high-quality, evidence-based protocol for the treatment of postoperative fatigue syndrome (POFS) after total joint arthroplasty (TJA) or fracture surgery with Chinese herbal medicine (CHM).

**Purpose:**

The purpose of this study was to explore the efficacy of CHM in the treatment of POFS after TJA or hip fracture surgery (HFS).

**Methods:**

We searched six databases to obtain randomized controlled trials (RCTs) of CHM for the treatment of POFS after TJA or HFS. The retrieval time limit was from the establishment of each database to August, 2022. According to the Cochrane Handbook for Systematic Reviews version 5.1, we used RevMan 5.3 to evaluate the quality of the studies. Stata 14.0 software was used to merge and analyze the data. The weighted mean difference (WMD) was the effect estimate for statistical analysis. We also performed subgroup analyses according to different types of surgeries.

**Results:**

A total of 11 RCTs were included in this study, comprising 430 cases in the CHM group and 432 cases in the control group (CG). The meta-analysis results showed that there was no significant difference in the Brief Profile of Mood States (BPOMS) score (WMD=0.08, 95% confidence interval (CI): -0.29 to 0.45, P=0.688), Christensen Fatigue scale (CHFS) score (WMD = 0.15, 95% CI: -0.09 to 0.39, P=0.214) or Identity-Consequence Fatigue Scale (ICFS) score (WMD=-0.40, 95% CI: -1.84 to 1.05, P=0.589) between the CHM group and the CG on the first postoperative day. The use of CHM significantly reduced the BPOMS score (WMD=-0.85 and WMD=-3.01, respectively), CHFS score (WMD=-1.01 and WMD= -1.45, respectively), and ICFS score (WMD=-3.51 and WMD=-5.26) on postoperative days 3 and 7. Compared with the CG, the CHM group had significantly increased serum transferrin and IgG levels on postoperative days 3 and 7. The subgroup analysis results suggested that the application of CHM in HFS patients improved fatigue symptoms on postoperative days 3 and 7, while the application of CHM to treat POFS in TJA patients had great inconsistency in the evaluation of different indicators.

**Conclusion:**

The application of CHM improved the fatigue status of POFS patients after TJA or HFS and increased the levels of transferrin and IgG in serum, which is conducive to promoting the postoperative rehabilitation process of patients. The subgroup analysis results showed that the application of CHM to intervene in POFS in HFS patients had obvious benefits.

## 1 INTRODUCTION

Postoperative fatigue syndrome (POFS) refers to a clinical syndrome in which patients have a series of symptoms, such as fatigue, depression, anxiety, insomnia, stress and inattention, after surgery, which seriously impair the quality of life and postoperative rehabilitation process of patients [1, 2]. POFS is a common complication after major surgery, which not only increases the inpatient burden of patients but is also not conducive to their recovery [3]. With aging, the prevalence of osteoarthritis, especially knee osteoarthritis and hip arthritis, continues to rise, which is associated with a heavy economic burden on the medical and health systems, patients, and society [4, 5]. Total joint arthroplasty (TJA), especially total hip arthroplasty (THA) and total knee arthroplasty (TKA), is the main treatment for end-stage bone and joint diseases [6, 7]. In particular, rehabilitation treatment after TJA or fracture surgery plays a key role in improving patients' postoperative satisfaction, improving quality of life and accelerating rehabilitation, and the occurrence of POFS undoubtedly hinders this process. Therefore, it is of great clinical value to explore an effective treatment for POFS after TJA or fracture surgery.

Notably, academic research on POFS started relatively late, and there is still a lack of research on POFS, especially on orthopedic surgery-related POFS. The pathogenesis and therapeutic targets of POFS are still unclear. At present, symptomatic treatment is the main treatment, and the therapeutic methods are limited. In recent years, Chinese herbal medicine (CHM), which can be applied in the form of decoctions, single herbal medicines, granules, capsules, *etc*., has been gradually used to treat PFOS. Miao *et al.* [8] showed that an herbal formula, Danggui Buxue decoction, can improve the fatigue symptoms of rats with fatigue syndrome, and its mechanism may be through interfering with the metabolism of ammonia, serine, and threonine. The effective components of single herbs, such as ginsenoside Rb1, have also been proven to improve the symptoms of fatigue in rats through the PI3K/Akt/Nrf2 signaling pathway [9]. According to this traditional Chinese medicine theory, CHM has a good clinical effect in the treatment of fatigue syndrome [10-12].

In recent years, the promotion and application of the concept of rapid rehabilitation have also indirectly encouraged bone surgeons to give importance to POFS. CHM is also used to treat POFS symptoms in patients after TJA or fracture surgery. However, there is no high-quality, evidence-based protocol for the treatment of POFS after TJA or fracture surgery with CHM; thus, surgeons and rehabilitation doctors may not be confident in employing this traditional herbal therapy. Therefore, we used a data synthesis method (meta-analysis) to provide an evidence-based evaluation of the efficacy and clinical application of CHM in the treatment of POFS after TJA or hip fracture surgery (HFS).

## 2 MATERIALS AND METHODS

The reporting of the implementation process and results of this study followed the Preferred Reporting Items for Systematic Reviews and Meta-Analyses (PRISMA) statement [13].

### 2.1 Inclusion and Exclusion Criteria

The inclusion criteria followed the population, intervention, comparison, outcomes, and study (PICOS) principle. 1) The study subjects were patients diagnosed with POFS after TJA (such as THA or TKA) or HFS. 2) The intervention measures in the treatment group were CHM but only Chinese herbal formulas and proprietary Chinese medicines. 3) The control group (CG) received routine treatment after the operation, such as anti-infective, anticoagulant, or analgesic drugs and rehabilitation exercises. 4) Outcome measures included primary outcome measures (Christensen Fatigue scale (CHFS), Brief Profile of Mood States (BPOMS), and Identity-Consequence Fatigue Scale (ICFS) scores) [14-16] and secondary outcome measures (transferrin (TRF), IgG, and Visual Analog Fatigue Scale (VAS-F) scores). 5) The study design was a randomized controlled trial (RCT). 6) The diagnostic criteria of POFS were clearly stated in the included studies.

The exclusion criteria were as follows: 1) duplicate publication of the same research data, 2) incomplete data, and 3) the study did not report at least one of the outcome indicators we were investigating.

### 2.2 Retrieval Strategy

We searched the Cochrane Library, PubMed/Medline, and EMBASE databases, the Chinese Biomedical Literature Database (CBM), and the China National Knowledge Infrastructure (CNKI) and Wanfang databases. The retrieval time was from the establishment of each database to August, 2022. The keywords of the search form constructed in this study were Chinese herbal medicine, medicinal plants, phytochemicals, total joint arthroplasty, total knee arthroplasty, total hip arthroplasty, fatigue syndrome, and fatigue. The retrieval was carried out by combining subject words with free words and was adjusted according to the characteristics of each database. In addition, we traced the references of the included studies to obtain more studies that met the inclusion criteria. The search formula for each database is shown in Annex 1.

### 2.3 Literature Screening and Data Extraction

Two evaluators independently screened the studies, extracted the data, and assessed the methodological quality according to the inclusion and exclusion criteria of this study. Any differences were resolved through discussion or consultation with the corresponding authors. Two evaluators independently extracted data from the final included studies. The extracted data included author, title, year of publication, sample size, type of surgery, intervention measures, study quality evaluation information, outcome indicators, and outcome measurement data.

### 2.4 Study Quality Evaluation

Methodological quality was evaluated using the risk of bias assessment tool in the Cochrane Handbook for Systematic Reviews version 5.1. The evaluation contents included the generation of random sequences, allocation concealment, selective reporting, blinding, data loss, and other types of bias. According to the standards of the evaluation manual, the study was examined as having a low risk, high risk, or unclear risk of bias.

### 2.5 Data Analysis

We employed the statistical software RevMan 5.3 provided by the international collaborative network of evidence-based medicine to evaluate the risk of bias in the included studies. Stata 14.0 software was used for data analysis of the outcome indicators. The outcome indicators of interest in this study were all continuous variables, so the weighted mean difference (WMD) was selected as the effect estimate for analysis, and the 95% confidence interval (CI) corresponding to the effect estimate was calculated. The models for merging the data of each outcome indicator were all random effects models. If there were outcome indicators that involved ≥ 10 included studies, a funnel chart was made to evaluate publication bias.

### 2.6 Subgroup Analysis

The types of surgery included in this study included TJA and HFS. Based on the above conditions, we conducted subgroup analysis according to different types of surgery (TJA or HFS), which helped us further subdivide the population of POFS patients to obtain more information on clinical value.

## 3 RESULTS

### 3.1 Search Results

We initially retrieved 1036 studies. After removing duplicate studies, reading the titles and abstracts, and reading the full texts, 11 RCTs were included in this study [14-24]. The specific retrieval process is shown in Fig. (**1**).

### 3.2 Basic Characteristics of the Included Studies

A total of 11 single-center RCTs [17-27] were included in this study. A total of 862 cases were included, including 430 cases in the CHM group and 432 cases in the CG. The sample size included in a single study ranged from 43 to 126 cases. The intervention measures in the CHM group were mainly Chinese herbal formulas and proprietary Chinese medicines, and these herbal medicines were administered mainly to replenish *Qi*, promote blood circulation, strengthen the spleen, and move *Qi*. The intervention measures in the CG were routine anti-infective, anticoagulant, and analgesic drugs and rehabilitation exercises. The types of surgery mainly included TJA (TKA or THA) and HFS. The basic characteristics of the included studies are mentioned in Table **1**.

### 3.3 Study Quality Evaluation

**Fig. (2):**
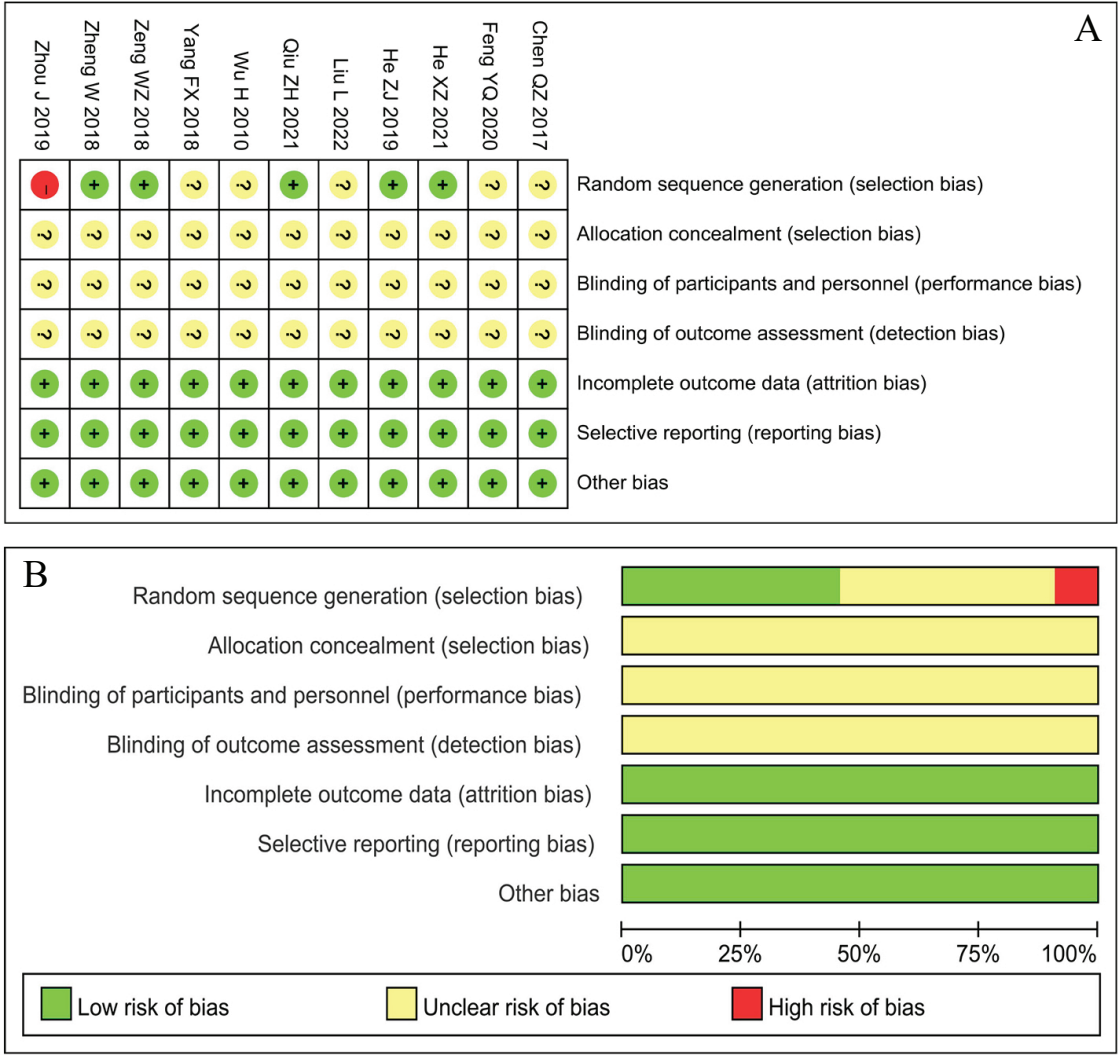
Risk of bias: (A) Quality assessment of individual studies. (B) Each risk of bias item is presented as a percentage across all included studies.

In terms of the use of randomization methods, 5 studies [18, 20, 22, 24, 27] used the random number table method, and five studies [17, 19, 22, 23, 25] only mentioned randomization methods but did not specify the specific method. In addition, one study [21] grouped cases according to the order of admission, which was judged as having a high risk of bias. None of the included studies [17-27] described allocation concealment or blinding and were judged as having an unclear risk of bias. The 11 included studies [17-27] had a low risk of bias in terms of incomplete data, selective reporting, and other risks. The overall quality of the included studies was acceptable (Fig. **2**).

### 3.4 Meta-Analysis

#### 3.4.1 Primary Outcomes

##### 3.4.1.1 BPOMS

A total of 5 studies [18, 19, 21-23] reported comparative data for the BPOMS. The results of the meta-analysis showed that there was no significant difference between the CHM group and the CG in the comparison of BPOMS scores on the first day after TJA or HFS (WMD=0.08, 95% CI: -0.29 to 0.45, *P*=0.688). On the 3^rd^ (WMD= -0.85, 95% CI: -1.35 to -0.35, *P*=0.001) and 7^th^ (WMD= -3.01, 95% CI: -5.86 to -0.16, *P*=0.039) days after the operation, the BPOMS score of the CHM group was lower than that of the CG (Table **2**).

Subgroup analysis by TJA or HFS showed that there was no significant difference in the BPOMS scores of the subgroups on the first day after the operation. On the third day after the operation, the CHM group had lower BPOMS scores for both TJA and HFS patients. In the BPOMS score comparison on the 7^th^ day after the operation, the HFS patients in the CHM group had improved postoperative BPOMS scores, but there was no significant difference for TJA patients compared to the CG (Table **2**).

###### 3.4.1.1.1 CHFS

A total of 5 studies [20, 21, 23-26] reported the comparison results of CHFS scores. In the comparison of the CHFS scores on the first day after the operation, there was no significant difference between the CHM group and the CG (WMD=0.15, 95% CI: -0.09 to 0.39, *p* = 0.214). In the comparison of the CHFS scores on the 3^rd^ day (WMD=-1.01, 95% CI:-1.94 to -0.09, *p* = 0.031) and the 7^th^ day (WMD=-1.45, 95% CI: -2.23 to -0.66, *p* <0.001), the CHFS scores in the CHM group were significantly lower than those in the CG (Table **2**).

The subgroup analysis results showed that there was no significant difference in the CHFS scores on the first day after the operation in the subgroups. In terms of improved CHFS scores of patients with HFS (the 3^rd^ and 7^th^ postoperative days) and TJA (the 7^th^ postoperative day), the CHM group had better results than the CG (Table **2**).

###### 3.4.1.1.2 ICFS

A total of 4 RCTs [18, 21, 23, 27] reported ICFS scores. The results of the meta-analysis showed that there was no significant difference between the two groups in the ICFS scores on the first day after the operation (WMD=-0.40, 95% CI: - 1.84 to 1.05, *p* = 0.589). In the comparison of ICFS scores between the two groups on the 3^rd^ day (WMD=-3.51, 95% CI:- 6.57 to -0.44, *p* = 0.025) and the 7^th^ day (WMD = -5.26, 95% CI: - 10.33 to -0.20, *p* = 0.042) after the operation, the CHM group had lower ICFS scores than the CG, and the difference was statistically significant (Table **2**).

The subgroup analysis results showed that there was no significant difference between the two groups on the 1^st^ or 3^rd^ days after the operation. In the comparison of ICFS scores on the 7^th^ day after the operation, the use of CHM for HFS patients showed better efficacy than the use of conventional therapy for patients in the CG, but there was no difference between the two groups among TJA patients (Table **2**).

### 3.5 Secondary Outcomes

#### 3.5.1 TRF

A total of 3 RCTs [21, 24, 25] reported TRF levels. On the 3^rd^ day (WMD=0.51, 95% CI: 0.22 to 0.81, *p* = 0.001) and 7^th^ day (WMD=0.21, 95% CI: 0.04 to 0.39, *p* = 0.018) after the operation, the serum TRF concentration of the CHM group was higher than that of the CG, and the difference was statistically significant. There were also significant differences in the subgroup analysis. In the comparison of TRF concentration on the first day after the operation, there was no significant difference in the use of CHM between HFS patients and the CG (Table **3**).

#### 3.5.2 VAS-F

A total of 4 studies [17, 18, 22, 23] reported VAS-F scores. Meta-analysis showed that there was no significant difference between the two groups in VAS-F scores on day 1 (WMD=-0.13, 95% CI: -0.49 to 0.23, *p* = 0.474) or day 7 (WMD=-1.20, 95% CI:- 2.56 to 0.15, *p* = 0.081) after the operation. In the comparison on the third day after the operation, the VAS-F score of the CHM group was lower than that of the CG, and the difference was statistically significant (WMD=-0.64, 95% CI: -1.00 to -0.28, *p* = 0.001) (Table **3**).

The subgroup analysis results showed that there was no significant difference in the subgroups on the first day after the operation. In the comparison of VAS-F scores on the 3^rd^ day after the operation, the difference between the two groups was statistically significant in the subgroup analysis. For the VAS-F score comparison on the 7^th^ day after the operation, CHM had better efficacy in HFS patients, but there was no difference in TJA patients (Table **3**).

#### 3.5.3 IgG

A total of 3 studies [18, 21, 23] reported the comparison of serum IgG concentrations. The meta-analysis results showed that there was no significant difference between the two groups in the comparison of IgG concentrations on the first day after the operation (WMD= 0.34, 95% CI: -0.03 to 0.70, *p* = 0.069). In the comparison of IgG concentrations on the 3^rd^ day (WMD=0.75, 95% CI: 0.40 to 1.10, *p* <0.001) and 7^th^ day (WMD=0.95, 95% CI: 0.59 to 1.30, *p* <0.001) after the operation, the CHM group had significantly higher serum IgG levels than the CG (Table **3**).

Subgroup analysis of CHM administration for TJA and HFS patients showed that IgG levels on the 3^rd^ and 7^th^ days after the operation were significantly different between the two groups (Table **3**).

### 3.6 Publication Bias Evaluation

Since no outcome measures included at least 10 studies, this study did not further develop a funnel chart to evaluate publication bias.

## 4 DISCUSSION

This study is the first systematic review and meta-analysis of the efficacy of CHM in the treatment of POFS after TJA or HFS and can provide evidence for the treatment of POFS with CHM. This study found that compared to the CG, there was no statistically significant difference in the BPOMS score, CHFS score, or ICFS score in the CHM group on the first day after the operation. The pathogenesis of POFS is complex and has not been fully elucidated. It is generally considered [9, 28] that immune system disorders, neuroendocrine disorders, and metabolic capacity decline occur after the body is injured by surgery, which leads to POFS [29, 30]. We think the best explanation is that trauma after TJA or HFS is very serious and that patients are still under the influence of narcotic drugs; their internal environmental balance, immune system, and stress protection ability are recovering, which may lead to insensitivity to external intervention. This possibility was also reflected in the changes in the secondary outcome indicators, TRF levels, VAS-F scores, and IgG levels. In terms of the improvement in the BPOMS score, CHFS score, and ICFS score on the third and seventh days after surgery, the combined application of CHM based on routine postoperative care significantly improved the fatigue symptoms of patients after TJA or HFS and the three different outcome indicators showed good consistency in the evaluation of the degree of fatigue. Similarly, the application of CHM significantly increased the levels of TRF and IgG in the serum of patients on the 3^rd^ and 7^th^ days after surgery, which plays an important role in enhancing immunity and improving the nutritional status of patients.

Through the analysis of the prescription and drug composition of Chinese patent medicine used in the CHM group, we found that the composition of botanical medicine is considered to invigorate *Qi*, activate blood circulation, strengthen the spleen, and move *Qi* in traditional Chinese medicine, which is consistent with the application law of CHM in the treatment of *Xulao*. Previous studies have found [8, 11, 31, 32] that components of CHM, such as Kaixin San, Danggui Buxue decoction, and Sijunzi decoction, can improve fatigue symptoms by affecting fatigue-related physiological markers, reducing oxidative damage, and enhancing immunity. These plant medicine components that have been proven to be potentially beneficial have the effects of invigorating *Qi*, activating blood circulation, strengthening the spleen, and moving *Qi*.

Subgroup analysis was carried out according to the type of surgery, TJA or HFS, and the subgroup analysis results indicated that there was no significant difference between the CHM group and CG in the comparison of the main outcome indicators on the first day after surgery. In addition, we found that in the comparison of different outcome indicators of patients with TJA, the difference between CHM and postoperative routine treatment did not always have the same positive effect, indicating that the use of CHM to treat POFS in TJA patients still shows great inconsistency in terms of different indicators. However, the use of CHM in HFS patients had a positive effect on the improvement of the BPOMS score, CHFS score, VAS-F score, and TRF and IgG levels on the 3^rd^ and 7^th^ days after the operation, indicating that the use of CHM to treat POFS in HFS patients has potential benefits. The conclusions of this study can provide guidance for clinicians in the treatment of POFS in TJA or HFS patients.

This study has the following limitations. First, CHM in this study included different types of herbal formulas and Chinese patent medicines, which may affect the extrapolation of the conclusions of this study. Second, the individuals included in this study were TJA or HFS patients. Although these two types of surgeries are large- and medium-sized bone surgeries, this may have also increased the heterogeneity between studies. Finally, the difference in POFS diagnostic criteria in different included studies may have impaired the reliability of the conclusions of this study. The resolution of the above limitations in future research will be conducive to providing higher-quality evidence-based information.

## CONCLUSION

This study found that the use of CHM can improve the degree of fatigue associated with POFS after TJA or HFS and increase the levels of TRF and IgG in serum, which will help clinicians have confidence in using CHM to treat POFS to promote the postoperative rehabilitation process of patients. The subgroup analysis results showed that the use of CHM to intervene in POFS in HFS patients had obvious benefits. Given the objective limitations of this study, future studies need to involve multicenter RCTs on the efficacy of specific types of CHMs to further determine their role in the treatment of POFS.

## Figures and Tables

**Fig. (1):**
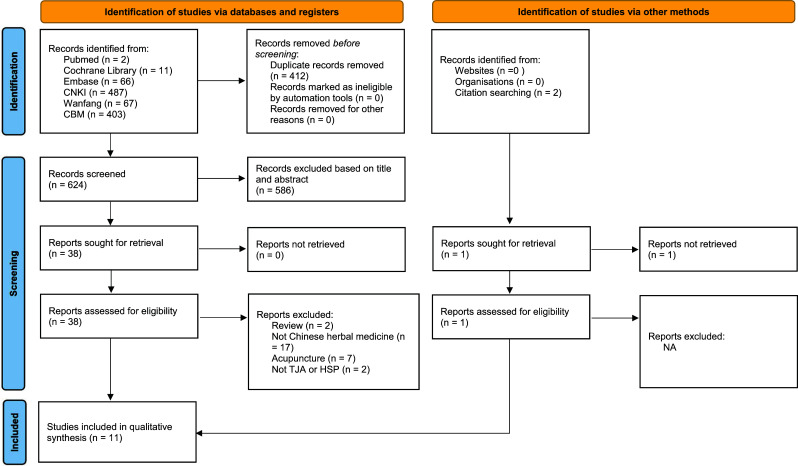
PRISMA flowchart of the selection process.

**Table 1:** Characteristics of 11 included RCTs.

**Study**	**Intervention**		**Type of Surgery**	**Sample Number (Male/Female)**		**Average Age (Years)**		**Follow-up**	**Endpoints**
	**CHM**	**CG**		**CHM**	**CG**	**CHM**	**CG**		
Liu *et al.*, 2022 [17]	*Wufu Jianxi* Prescription + CG	RPOT	TKA	23 (7/16)	20 (5/15)	80.3±8.6	78.9±8.6	3 days	BPOMS, VAS-F
Qiu *et al.*, 2021 [18]	*Bazhen* Decoction + CG	RPOT	THA	31 (8/23)	32 (7/25)	72.84±5.05	70.97±6.12	7 days	BPOMS, VAS-F, ICFS, IgG
Feng *et al.*, 2020 [19]	*Hongjingtian* Capsules + CG	RPOT	THA	63 (32/31)	63 (33/30)	49.92±7.57	50.86±9.11	3 weeks	BPOMS
He Z *et al.*, 2019 [20]	*Jianpi Yiqi Huoxue Huayu* Prescription + CG	RPOT	TKA	25 (5/20)	28 (8/20)	66.32±8.14	67.75±7.7	6 days	CHFS
Zhou *et al.*, 2019 [21]	*Shudi Zhuanggu* Prescription + CG	RPOT	THA	60 (25/35)	61 (27/34)	70.68±5.05	69.69±5.04	2 weeks	CHFS, BPMOS, ICFS, TRF, IgG
Yang *et al.*, 2018 [22]	*Hongjingtian* Capsules + CG	RPOT	THA	57 (30/27)	57 (28/29)	44.92±7.90	46.81±9.72	2 weeks	BPOMS, VAS-F
Zheng *et al.*, 2018 [23]	*Bazhen* Decoction + CG	RPOT	HFS	28 (11/17)	28 (13/15)	79.86±7.89	77.57±9.21	7 days	BPOMS, VAS-F, ICFS, IgG
Zeng *et al.*, 2018 [24]	*Buqi Jianpi Huoxue* Prescription + CG	RPOT	HFS	55 (19/36)	55 (20/35)	72.35±3.44	72.15±3.26	7 days	CHFS, TRF
Chen *et al.*, 2017 [25]	*Taohong Buzhong Yiqi* Decoction + CG	RPOT	HFS	30 (14/16)	30 (12/18)	71.23±8.23	72.50±7.36	7 days	CHFS, TRF
Wu *et al.*, 2010 [26]	Jianpi Huoxue Prescription + CG	RPOT	HFS	28 (-/-)	28 (-/-)	NR	NR	7 days	CHFS
He X *et al.*, 2021 [27]	*Yiqi Huoxue* Prescription + CG	RPOT	HFS	30 (12/18)	30 (10/20)	77.57±8.41	75.17±9.69	6 days	ICFS

**Abbreviations:** RCTs: randomized controlled trials; CHM: Chinese herbal medicine; CG: control group; RPOT: Routine Postoperative Treatments; HFS: hip fracture surgery; THA: total hip arthroplasty; TKA: total knee arthroplasty; NR: not reported; CHFS: Christensen fatigue scale; BPOMS: brief profile of mood states; TRF: transferrin; VAS-F: visual analogic scale of fatigue; ICFS: identity-consequence fatigue scale.

**Table 2:** Meta-analysis results of primary outcomes.

**Outcome or Subgroup Title**	**No. of Studies**	**No. of Participants**	**I^2^ (%)**	**WMD (95% CI)**	***p***
BPOMS (POD 1)	3	240	0	0.08 (-0.29, 0.45)	0.688
TJA	2	184	0	0.15 (-0.247, 0.55)	0.457
HFS	1	56	NA	-0.39 (-1.38, 0.60)	0.440
BPOMS (POD 3)	4	283	37.5	-0.85 (-1.35, -0.35)	0.001
TJA	3	227	34.8	-0.74 (-1.26, -0.22)	0.006
HFS	1	56	NA	-1.32 (-2.34, -0.30)	0.011
BPOMS (POD 7)	5	480	97.9	-3.01 (-5.86, -0.16)	0.039
TJA	4	424	98.5	-3.31 (-7.10, 0.47)	0.086
HFS	1	56	NA	-1.82 (-2.84, -0.80)	<0.001
Christensen (POD 1)	3	234	0	0.15 (-0.09, 0.39)	0.214
TJA	2	174	0	0.13 (-0.13, 0.39)	0.334
HFS	1	60	NA	0.27 (-0.32, 0.86)	0.372
Christensen (POD 3)	4	290	93.8	-1.01 (-1.94, -0.09)	0.031
TJA	2	178	97.9	-1.44 (-3.59, 0.71)	0.188
HFS	2	112	0	-0.59 (-0.97, -0.20)	0.003
Christensen (POD 7)	4	347	93.4	-1.45 (-2.23, -0.66)	<0.001
TJA	1	121	NA	-0.39 (-0.71, -0.07)	0.017
HFS	3	226	33.7	-1.74 (-2.06, -1.42)	<0.001
ICFS (POD 1)	4	300	22.4	-0.40 (-1.84, 1.05)	0.589
TJA	2	184	0	-0.24 (-1.27, 0.80)	0.652
HFS	2	116	67.9	-0.96 (-6.86, 4.93)	0.748
ICFS (POD 3)	4	300	84.5	-3.51 (-6.57, -0.44)	0.025
TJA	2	184	91.1	-2.92 (-6.73, 0.88)	0.133
HFS	2	116	64.6	-4.27 (-9.91, 1.38)	0.139
ICFS (POD 7)	3	240	96.6	-5.26 (-10.33, -0.20)	0.042
TJA	2	184	98.1	-4.53 (-10.98, 1.93)	0.170
HFS	1	56	NA	-6.93 (-10.21, -3.65)	<0.001

**Abbreviation:** WMD: weighted mean difference; POD: postoperative day; HFS: hip fracture surgery; TJA: total joint arthroplasty; CHFS: Christensen fatigue scale; BPOMS: brief profile of mood states; ICFS: identity-consequence fatigue scale; NA: not applicable.

**Note:** † The forest plots of the primary outcome indicators are shown in Annex 2.

**Table 3:** Meta-analysis results of secondary outcomes.

**Outcome or Subgroup Title**	**No. of Studies**	**No. of Participants**	**I^2^ (%)**	**WMD (95% CI)**	***P***
TRF (POD 1)	1	60	NA	-0.30 (-0.81, 0.21)	0.250
HFS	1	60	NA	-0.30 (-0.81, 0.21)	0.250
TRF (POD 3)	2	181	17.1	0.51 (0.22, 0.81)	0.001
TJA	1	121	NA	0.40 (0.04, 0.76)	0.030
HFS	1	60	NA	0.75 (0.23, 1.28)	0.005
TRF (POD 7)	3	291	97.3	0.21 (0.04, 0.39)	0.018
TJA	1	121	NA	0.07 (0.01, 0.13)	0.016
HFS	2	170	94.3	0.29 (0.14, 0.43)	<0.001
VAS-F (POD 1)	2	119	65.4	-0.13 (-0.49, 0.23)	0.474
TJA	1	63	NA	0.16 (-0.33, 0.65)	0.526
HFS	1	56	NA	-0.47 (-1.00, 0.06)	0.083
VAS-F (POD 3)	3	162	15.3	-0.64 (-1.00, -0.28)	0.001
TJA	2	106	26.4	-0.51 (-0.92, -0.10)	0.015
HFS	1	56	NA	-0.89 (-1.43, -0.35)	0.001
VAS-F (POD 7)	3	233	97.8	-1.20 (-2.56, 0.15)	0.081
TJA	2	177	98.7	-1.44 (-3.23, 0.35)	0.116
HFS	1	56	NA	-0.71 (-1.25, -0.17)	0.011
IgG (POD 1)	2	119	45.3	0.34 (-0.03, 0.70)	0.069
TJA	1	63	NA	0.11 (-0.39, 0.60)	0.675
HFS	1	56	NA	0.61 (0.07, 1.15)	0.026
IgG (POD 3)	3	240	0	0.75 (0.40, 1.10)	<0.001
TJA	2	184	0	0.62 (0.21, 1.02)	0.003
HFS	1	56	NA	1.13 (0.44, 1.82)	0.001
IgG (POD 7)	3	240	0	0.95 (0.59, 1.30)	<0.001
TJA	2	184	0	0.89 (0.48, 1.31)	<0.001
HFS	1	56	NA	1.10 (0.41, 1.79)	0.002

**Abbreviation:** WMD: weighted mean difference; POD: postoperative day; HFS: hip fracture surgery; TJA: total joint arthroplasty; TRF: transferrin; VAS-F: visual analogic scale of fatigue; NA: not applicable.

**Note:** † The forest plots of the secondary outcome indicators are shown in Annex 3.

## Data Availability

The authors declare that the materials described in the manuscript, including all relevant raw data, will be freely available to any scientist wishing to use them for non-commercial purposes without breaching participant confidentiality.

## LIST OF ABBREVIATIONS

BPOMS: Brief Profile of Mood States
CBM: Chinese Biomedical Literature Database
CG: Control Group
CI: Confidence Interval
CNKI: China National Knowledge Infrastructure
CHFS: Christensen Fatigue scale
HFS: Hip Fracture Surgery
ICFS: Identity-Consequence Fatigue Scale
PRISMA: Preferred Reporting Items for Systematic Reviews and Meta-Analyses
POFS: Postoperative Fatigue Syndrome
RCT: Randomized Controlled Trial
TJA: Total Joint Arthroplasty
WMD: Weighted Mean Difference

## STANDARDS OF REPORTING

PRISMA guidelines and methodology were followed.

## SUPPLEMENTARY MATERIAL

PRISMA checklist is available as supplementary material on the publisher’s website along with the published article.
